# Community-based educational interventions for prevention of type II diabetes: a global systematic review and meta-analysis

**DOI:** 10.1186/s13643-021-01619-3

**Published:** 2021-03-20

**Authors:** Tayebeh Shirvani, Zeinab Javadivala, Somayeh Azimi, Abdolreza Shaghaghi, Zahra Fathifar, H. D. R. Devender Bhalla, Mohammadhiwa Abdekhoda, Haidar Nadrian

**Affiliations:** 1 Department of Health Education and Promotion, Faculty of Health, Tabriz University of Medical Sciences, Tabriz, Iran; 2 Faculty of Health, Tabriz University of Medical Sciences, Tabriz, Iran; 3 Iranian Epilepsy Association, Tehran, Iran; 4 Pôle Universitaire Euclide Intergovernmental UN Treaty 49006/49007, Bangui, Central African Republic; 5 Department of Health Informatics, Faculty of Health Informatics and Management, Tabriz University of Medical Sciences, Tabriz, Iran; 6 Social Determinants of Health Research Center, Tabriz University of Medical Sciences, Tabriz, Iran

**Keywords:** Educational intervention, Community-based, Behavior change, Diabetes, Prevention, Epidemiology

## Abstract

**Purpose:**

Our objective was to estimate the change in community-based education interventions throughout the world that may effectuate in risk parameters of type II diabetes (T2D), including the diabetes incidence rate, fasting blood glucose, hemoglobin A1C, body mass index, waist circumference, and systolic and diastolic blood pressure.

**Methods:**

A comprehensive search for globally eligible studies was conducted on PubMed, Embase, ProQuest, CINAHL nursing & allied health source, Cochrane Library, Google Scholar, conference proceedings, and reference lists. Data were extracted using JBI standardized data extraction tool. The primary outcome variables were diabetes incidence rate, fasting blood sugar (FBS), hemoglobin A1c (HbAlc), body mass index (BMI), waist circumference (WC), systolic/diastolic blood pressure (s/d BP). Random-effects meta-analysis and sub-group analyses were conducted.

**Results:**

Nineteen interventional studies were included in the review, and ten studies were pooled in the meta-analysis (*n* = 16,106, mean age = 41.5 years). The incidence rate of T2D was reported in three trials, within which the risk of developing T2D was reduced by 54.0% in favor of community-based educational interventions, (RR = 0.54, 95% CI = 0.38–0.75; *p* < 0.001). In eleven (*n* = 11,587) and six (*n* = 6416) studies, the pooled mean differences were − 0.33 (95% CI: − 0.45 to − 0.20, *p* < 0.0001) and − 0.15 (95% CI: − 0.28 to − 0.03, *p* < 0.0001) for FBS and HbA1c levels, respectively. Positive significant effects were observed on reducing BMI [pooled mean difference = − 0.47 (95% CI: − 0.66 to − 0.28), *I*^2^ = 95.7%, *p* < 0.0001] and WC [pooled mean difference = − 0.66 (95% CI: − 0.89 to − 0.43), *I*^2^ = 97.3%, *p* < 0.0001]. The use of theoretical frameworks was found to provide a 48.0% change in fasting blood sugar.

**Conclusions:**

Based on a comprehensive data collection of about 16,106 participants and reasonable analyses, we conclude that educational interventions may reduce diabetes incidence by 54.0%, particularly through reductions in fasting blood glucose, body mass index, and waist circumference. The diabetes risk parameters may favorably improve irrespective of the duration of intervention, at as low as 6 months. The application of theoretical frameworks while designing educational interventions is also encouraged.

**Systematic review registration:**

PROSPERO CRD42018115877

**Supplementary Information:**

The online version contains supplementary material available at 10.1186/s13643-021-01619-3.

## Introduction

Type-II Diabetes Mellitus (hereafter referred to as diabetes) is the most prevalent form of diabetes, and about 422 million adults are affected, worldwide [1]. In some regions, such as the Middle-East and North Africa [2] (MENA) countries, including Iran [2, 3], the burden of diabetes has been on the rise [4]. The continual rise in the burden of diabetes is perplexing because countries, since long, have national public education and prevention programs on diabetes [5].

To have an effective and adequate intervention for the prevention of diabetes, the fulfilment of at least three conditions is suggested. First, the interventions address the public at large, such as through public education on diabetes. Second, the use of prescribed theoretical frameworks while designing interventions [6]. Third, the interventions should address the majority of underlying pathogenic mechanisms. Public education is considered a powerful tool in the primary prevention of diabetes [7]. Public health knowledge is an important component of health literacy, which bears significance in improving the prevention of diseases [6]. The public’s health education can also improve one’s cognitive levels, unhealthy attitude and behaviors, prevention awareness, and acceptance of scientific concepts [7]. Advocacy agencies, such as the International Diabetes Federation, also suggests self-care measures (such as following a healthy diet) for optimal control of blood glucose [8]. In the absence of adequate self-care behaviors, there is a risk of premature mortality and complications among those with diabetes [9]. Therefore, community-based educational programs may be helpful in addressing various aspects of the patients’ health status including health behaviors, diet and physical activity [10], and quality of life [11] as well. Such programs show promise in T2DM prevention [12], as they can reach general populations that are not included in conventional healthcare settings, and usually target various groups within a community [13]. They may be also helpful in identifying the protective factors within different cultures, which may lead to provision of more support for the patients within the community [14].

Similarly, interventions are an embodiment of theories [15]. Theories help to make sense of complex phenomena by providing tentative explanations of the reasons and circumstances behind particular behaviors. Interventions can then target those behaviors. Others have also shown that adhering to a rigid theoretical framework is both recommended [6] and beneficial [16] because theoretically informed interventions lead to better outcomes. As an example, the health belief model [17] is useful in explicating self-care activities such as diabetes management recommendations and has a focus on behavior related to the prevention of disease.

Several current systematic reviews [11, 13, 18] have reported the positive effects of community-based programs on diabetes prevention. Obviously, a significant number of community-based programs may not involve educational interventions aiming at lifestyle change [13]. In previous systematic reviews and meta-analyses [11, 13, 18], however, no differentiation was considered between the community-based interventions with or without educational program. There is also a scarcity in the consolidated data describing the outcomes of community-based educational interventions, and in particular by theoretical framework, considerations on community factors, and intervention delivery method. It is also unknown that which behavior change theories and models are more practical and useful in such interventions. In other words, to the best of our knowledge, there has not been any globally focused systematic analysis of community-based interventional outcomes related to the prevention of diabetes through the necessary simultaneous perspective of public education and theoretical frameworks. Thus, the primary objective of our study was to estimate the change in community-based education interventions throughout the world that may effectuate in risk parameters of diabetes, including diabetes incidence rate, fasting blood glucose, and hemoglobin A1c. Our secondary objectives were to estimate any possible change in secondary diabetes risk parameters, including body weight, body mass index, waist circumference, and systolic and diastolic blood pressure. Moreover, we tried to determine the role that gender, age, duration of follow-up, and the use of a theoretical framework may play in conveying a change in diabetes risk. We believe our work would provide a comprehensive summary of available research to help have better anti-diabetes interventions and policies for safeguarding the health of the communities throughout the world.

## Methods

### Eligibility criteria

#### Participants and types of interventions

The eligibility criteria for our review were all community-based interventional studies with either randomized or non-randomized control group designs implemented on the general populations and/or the participants at-risk for diabetes within communities with a geographical focus on global articles. Another criterion was the studies that investigated the effectiveness of community-based educational strategies (focused on self-care behaviors change, lifestyle change, physical activity, and individual behavior change). Our exclusion criterion was those studies which used pharmacological treatment within their effectuated interventions.

Community-based intervention was defined as those programs that target the entire population living within a specific location outside clinical/healthcare settings. The theoretical framework was defined as a disease prevention model based on any established educational theory [17]. The criteria for the theoretical framework were intentionally kept broad enough to include all possible aspects of educating the public. The comparator was no intervention or standard treatment for the control group. The studies that included samples with metabolic syndrome and gestational diabetes mellitus were excluded. As the focus of our study was on diabetes prevention, we included the interventions that conducted only on general populations and/or the participants at-risk for diabetes within communities. So, the studies conducted on the diagnosed patients with diabetes types I and II were also excluded.

#### Outcome measures

The studies should have measured the following parameters: diabetes incidence rate, fasting blood sugar (FBS), hemoglobin A1c (A1C) or 2-h postprandial glucose (PPG) and/or change in mean BMI, the incidence of obesity, and behavioral outcomes (physical activity, diet control, self-care behaviors, quality of life, or other suitable ecological factors).

### Search strategy

To search for studies, we included Medical Subject Headings (MeSH) terms related to diabetes prevention and community-based educational and behavioral interventions (Suppl file 1). Initially, we conducted a limited search of MEDLINE and Embase. All studies published in English after 2000 till 2020 were included. Then, we undertook an analysis of the text words contained in the title and abstract and on the index terms used to describe the words. As the second step, we searched all identified keywords and index terms across the following databases: PubMed, Embase, ProQuest, CINAHL nursing & allied health source, and Cochrane Library. We also searched Google Scholar for additional studies and conference papers. We also hand-searched the reference list of all articles to identify any possible additional studies. In case the data of interest was not reported in studies, we tried to contact the authors for missing information or compute it based on the pre/post-intervention values reported.

### Data extraction and management

The initial eligibility was assessed based on the study’s title and abstract, and the data were extracted using JBI standardized data extraction tool and a standardized data extraction sheet designed for this study (Suppl file 2). We considered diabetes incidence rate (the likelihood and relative risk of developing diabetes) and glucose outcomes, i.e., FBS, A1C, or 2-h PPG as primary outcomes of interest. Therefore, among those included studies that reported the outcomes, we extracted the number of samples who developed diabetes at the end of the intervention, as well as the pretest/posttest (pre/post) changes in FBS (mmol/L), A1C (percentage points and mmol/mol), and PPG (mmol/L). We also extracted the pre/post changes in body weight (kg), body mass index (BMI), waist circumference, and systolic/diastolic blood pressure. We performed a subgroup analysis based on age group, gender, theoretical framework, and duration of follow-up.

### Methodological quality and risk of bias

Similar to previous meta-analysis studies [18–20] on community-based interventions, we assessed the quality of eligible studies by applying the following quality indicators: First, *the outcome used:* blood glucose (2 points), self-reported risk factors (1 point), and anthropometric measurements alone (1 point). Second, *attrition:* 20% (2-points), 20–40% (1 point), different attrition between study arms (0 point). Third, *analysis technique:* intent-to-treat (2 points), per protocol (1 point). Fourth, *external validity:* interventions described sufficiently in terms of program description (1 point), the qualification of intervention delivery agents (1 point), costs and resources used to deliver the program (1 point), and the acceptability of the program among participants and/or providers (1 point) to allow for external reproducibility. All included studies were scored on a 10-point scale and were categorized as of low (≤ 5), medium (6–7), or high (≥ 8) quality. The risk of bias in the inclusion of studies was assessed independently by two authors (TS and SA), and a third reviewer (HN) resolved any disagreement.

### Statistical analysis

To assess small study bias, we calculated Egger’s test score and used funnel plots. In the case of positive Egger’s test, we conducted a metatrim analysis and calculated an adjusted effect size.

The mean differences (MDs) and 95% confidence intervals (CIs) were calculated using StataCorp Stata 14.2 software. The MD divided by the study’s standard deviation was used to create an index, the standardized MD that would be comparable across the studies. Concerning meta-analysis, we pooled our quantitative data and the results were expressed in terms of the weighted mean difference. In the case where the statistical pooling was not possible, we presented our findings in narrative form using tables and figures, as appropriate. We used the Cochrane Q test to examine the between-study heterogeneity using *I*^2^ values and tau^2^ of between-study variance.

## Results

### Bibliometric results

**Fig. 1:**
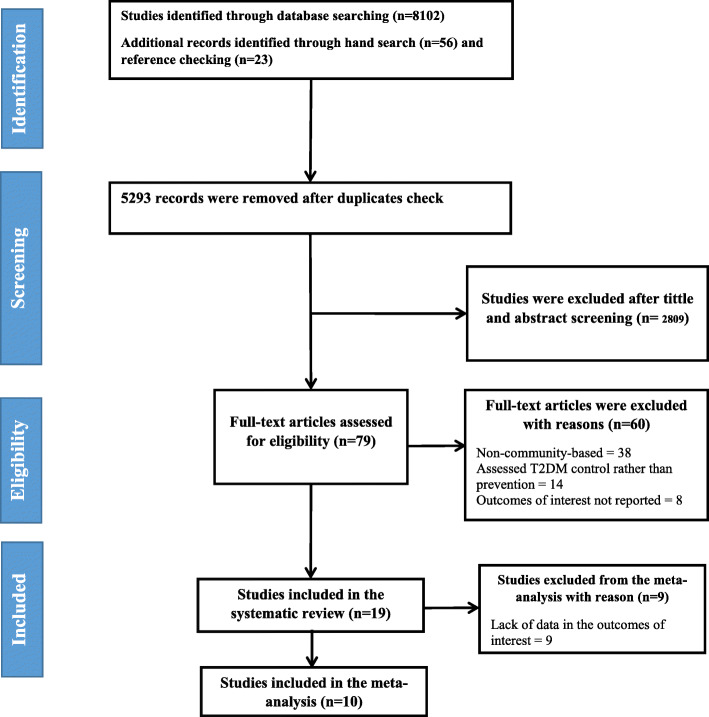
Flowchart describing the pathway to search of community-based studies that had education as an intervention for the prevention of diabetes

We searched through a total of 8181 records from various bibliometric sources, including 56 hand-searched items. Of these, we found a total of 19 studies that matched our inclusion criteria, (Fig. 1 and Suppl file 2), thus becoming eligible for our systematic review. Of these 19 studies, eight and six studies were adjudged to be of medium and high qualities, respectively, while five studies were found to be of low quality (Table 1). Twelve studies were found with lacks in data of interest. After contacting the authors for missing information, the authors of three studies provided us with complementary data. So, from among a total of 19 studies, we included a total of ten studies with randomized controlled design into the meta-analysis. The remaining nine studies were not included in the analysis due to the lack of their full reports.

**Table 1:** Quality assessment indicators of community-based studies in diabetes that had education as their intervention for the prevention of diabetes

Reference	Outcome	Attrition	Analysis technique	External validity				Overall study quality rating
				Program description	Qualification of intervention delivery agents	Costs and resources	Acceptability of the program	
**Balagopal et al.** [21]	Yes	Yes	Unclear	Yes	No	No	Yes	Medium
**Ibrahim et al.** [22]	Yes	Yes	Unclear	Yes	Yes	Unclear	Unclear	Medium
**Rowan et al.** [23]	Yes	No	Unclear	Yes	Yes	No	No	Low
**Penn et al.** [24]	No	Yes	No	Yes	Yes	No	Yes	Medium
**Katula et al.** [25]	Yes	Yes	Unclear	Yes	No	No	Yes	Medium
**Ackermann et al.** [26]	No	No	Unclear	Yes	No	Unclear	No	Low
**Ockene, et al.** [27]	Yes	Yes	Unclear	Yes	Yes	Unclear	Yes	High
**Daniel et al.** [28]	Yes	No	No	Yes	No	No	Unclear	Low
**Raman et al.** [29]	Yes	No	Unclear	Yes	Yes	Unclear	No	Low
**Balagopal et al.** [30]	Yes	Unclear	Yes	Yes	No	Unclear	Yes	Medium
**Ramachandran et al.** [31]	Yes	Unclear	Yes	Yes	Yes	Yes	Yes	High
**Harati et al.** [32]	Yes	No	Yes	Yes	Yes	Yes	Yes	High
**D.P.P.R Group** [33]	Yes	Yes	Yes	No	Unclear	Yes	Yes	High
**Davies et al.** [34]	Yes	No	Yes	Yes	Yes	Yes	Yes	High
**Yin et al.** [35]	Yes	Yes	Yes	Yes	No	Unclear	Yes	High
**Pedley et al.** [36]	Yes	Yes	Unclear	No	No	Unclear	Yes	Medium
**Sranacharoenpong et al.** [37]	Yes	No	Unclear	Yes	No	No	Yes	Medium
**Soltero et al.** [38]	Yes	Unclear	Unclear	Yes	No	Yes	Yes	Medium
**Soltero et al.** [39]	No	Unclear	Unclear	Yes	Yes	Unclear	Yes	Low

*DPPC* Diabetes Prevention Program Coordinating Centre

The majority (*n* = 12, 63.1%) of the 19 studies were conducted in high-income countries [23–30, 33, 34, 36, 38, 39], but more importantly, in merely three of them including nine studies in the USA, two in the UK, and one in Canada. The remaining seven studies were conducted in upper- and lower-middle-income countries [22, 32], including three in India and one each in China, Thailand, Iran, and Malaysia (Suppl file 2). These studies had a total of 16,106 participants (7391 cases and 9715 controls). Of 19 studies, 13 studies were conducted in urban and suburban areas [22–27, 29, 32–34, 36, 38, 39], four in rural areas [21, 28, 30, 35], and two in both kinds [37].

Ten studies were randomized [25–28, 32–35, 37, 38], five had a pre–post design, [21, 23, 24, 30, 38], while two studies were non-randomized [22, 29] (Supplementary file 2). Thirteen studies had adults alone [22–27, 30, 32–37], one had both children and adults [21], while two studies had children alone [29, 38]. The additional descriptive data of these studies are provided in Supplementary file 2. A theoretical framework was applied in only eight out of 19 studies. The frameworks used in the studies were social cognitive theory in four studies [27–29, 38], t5 instructional design in two studies [37, 39], and health belief [22] and social marketing [24] theories in one study each. In terms of educational interventions, 12 studies were aimed at improving lifestyle and increasing physical activity.

**Fig. 2:**
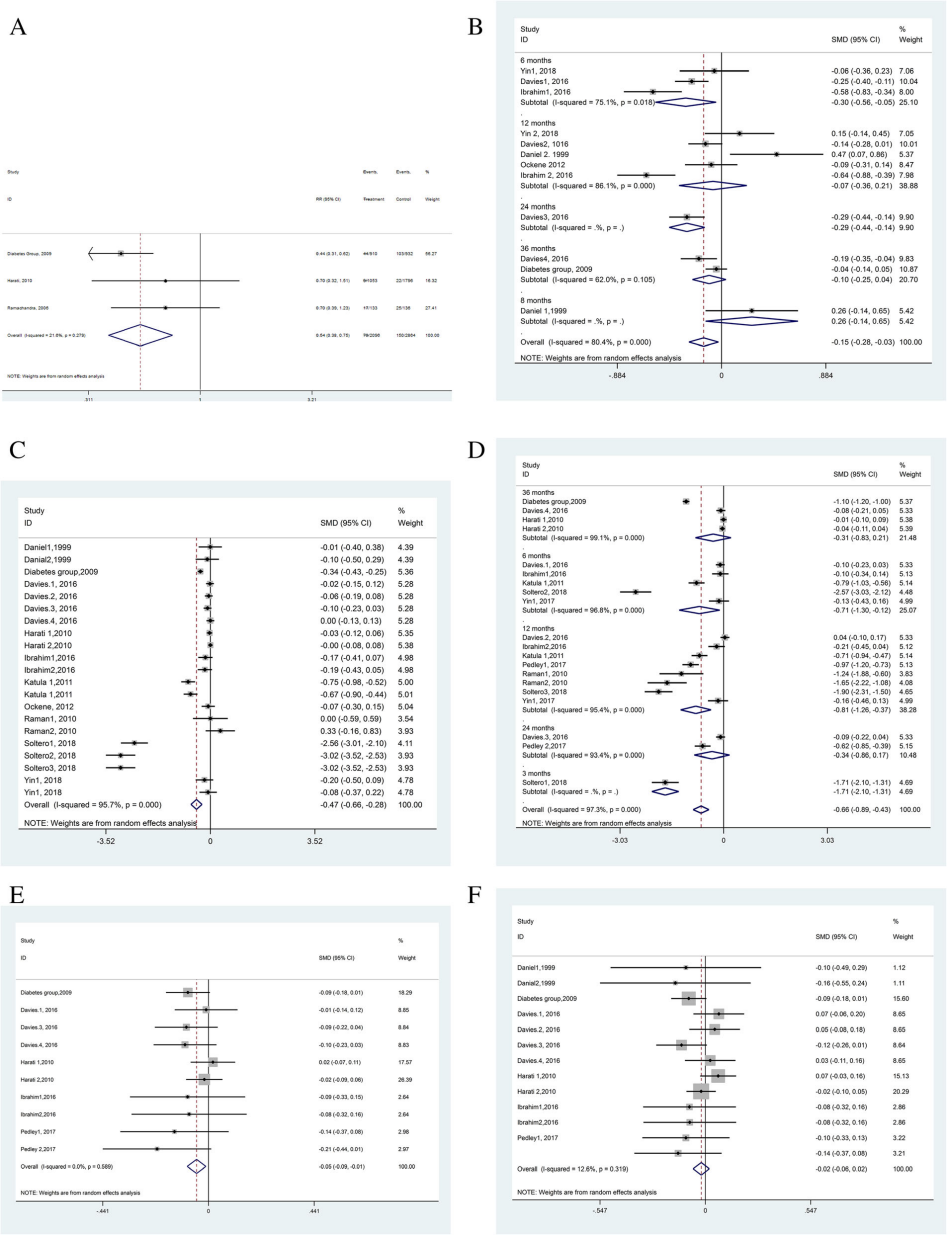
Meta-analysis results showing the effect of diabetes interventions on diabetes incidence (**a**), HBA1c level (**b**), body mass index (**c**), waist circumstance (**d**), and diastolic (**e**) and systolic (**f**) blood pressure

**Fig. 3:**
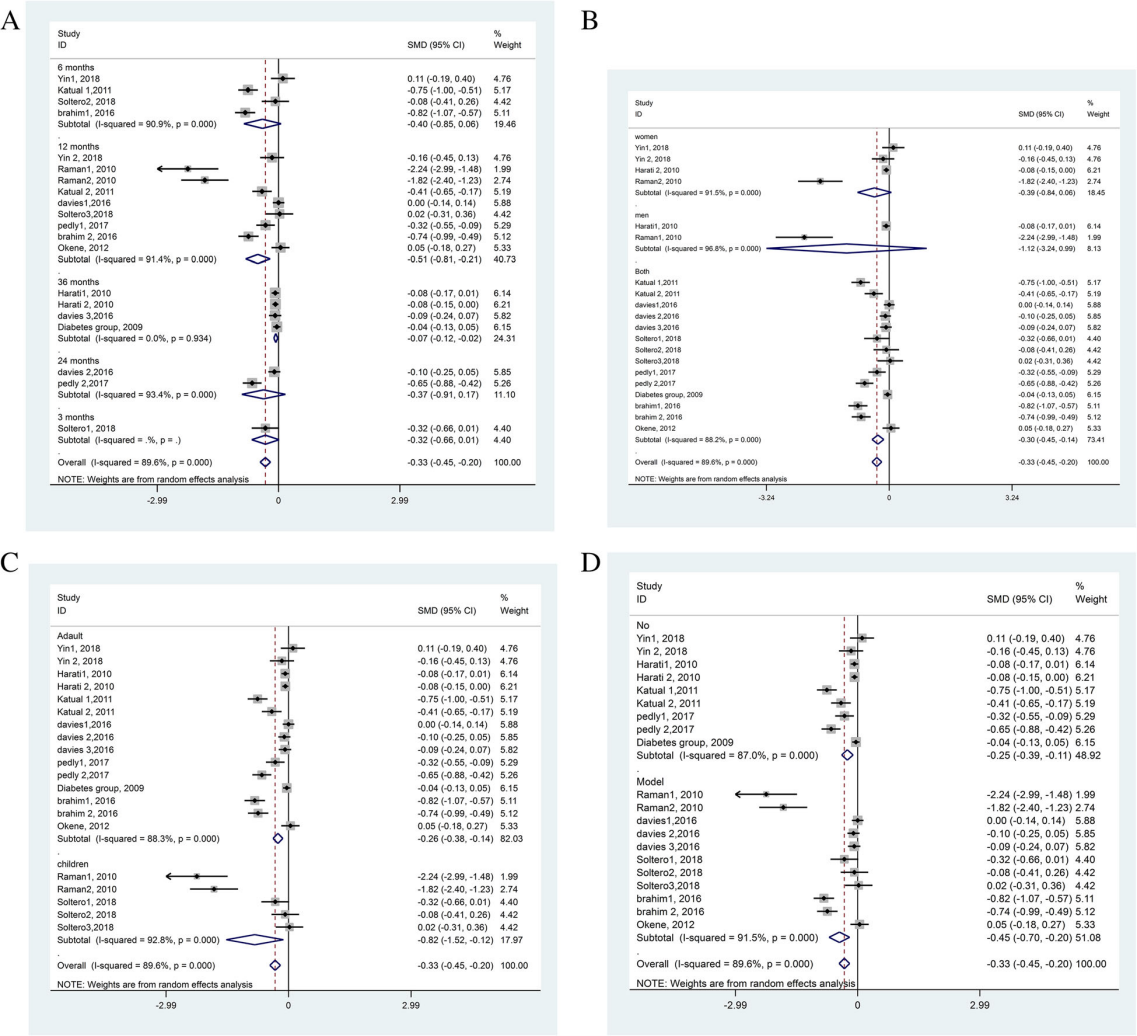
Subgroup meta-analysis results showing the effect of diabetes interventions on fasting blood glucose levels based on the duration of follow-up (**a**), gender (**b**), age (**c**), and using theoretical framework for designing their interventions (**d**)

**Fig. 4:**
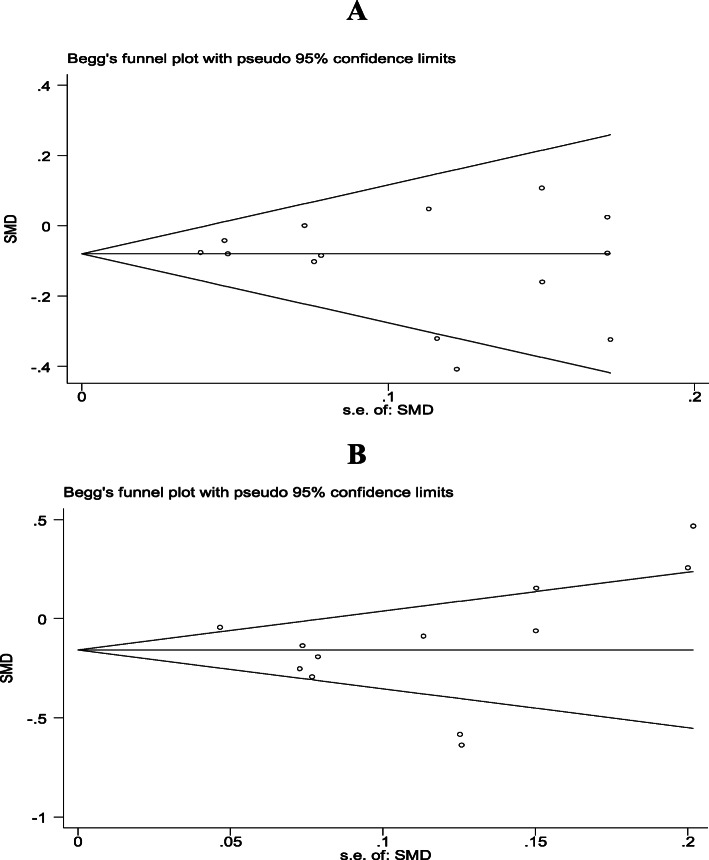
Funnel plots showing the effects of heterogeneity and publication bias in studies that addressed the prevention of diabetes through interventions for effecting change in the fasting blood glucose (**a**) and hemoglobin A1c (**b**) levels

Regarding meta-analyses, after excluding studies with large effect size [29, 39], upon sensitivity analysis, we did not find a decrease in the heterogeneity of results (Figs. 2–3). Also, the funnel plot did not show any evidence of publication bias, and the Egger test was not significant at 5.0% (Fig. 4).

### Diabetes results

The incidence rate of diabetes was reported in only three studies [13, 31, 32], within which one was a cluster-randomized study [32]. These studies reported the incidence of diabetes at 30 and 36 months of following up 2096 individuals. Upon pooling the effects, we found that among those in the intervention groups, 70 out of 2096 cases developed diabetes. However, in the comparator groups, 150 out of 2864 individuals developed diabetes over the same follow-up periods. Thus, the calculated absolute effect of the interventions was 29 fewer individuals per 1000 (95% CI 18–41). Overall, the risk of developing diabetes was 46.0% lower in the community-based educational interventions, compared with the control groups (RR = 0.54, 95% CI = 0.38–0.75; *p* < 0.001), the absolute risk reduction (Risk Difference) was 3.8% (RD = .038, 95% CI = .114–.037; *p* < 0.001) (Fig. 2a).

With regards to glycemic control, a total of 11 (*N* = 18,004, cases = 11,587, 64.3%) and six (*N* = 9471, cases = 6416, 67.7%) studies assessed the effect of their interventions on reducing FBS and A1c levels, respectively. The pooled difference in the mean FBS and A1c levels was − 0.33 (95% CI − 0.45 to − 0.20, *p* < 0.0001) and − 0.15 (95% CI − 0.28 to − 0.03, *p* < 0.0001) (Figs. 2b and 3), respectively.

The effect of interventions on reducing secondary parameters, including BMI, WC, sBP, and dBP levels were evaluated in ten, nine, six, and five studies, respectively. The total number of participants for evaluation of these parameters were 19,491 (cases = 12,555, 64.4%), 19,637 (cases = 12,637, 64.3%), 17,867 (cases = 11,493, 64.3%), and 17,404 (cases = 11559, 66.4%), respectively. The pooled mean difference was − 0.47 (95% CI − 0.66 to − 0.28, *I*^2^ = 95.7%, *p* < 0.0001) and − 0.66 (95% CI − 0.89 to − 0.43, *I*^2^ = 97.3%, *p* < 0.0001), indicating a positive effect on reducing BMI and WC alone (Fig. 2c–f), respectively. There was no favorable effect of effected interventions on reducing sBP and dBP.

We also performed various subgroup analyses based on the duration of follow-up, gender, age, and theoretical framework vis-à-vis a change in the FBS levels (Figs. 1, 2, 3, 4). These results suggested that the standardized mean difference in FBS did not seem to depend upon the duration of follow-up. This was so because, while at 12 months follow-up, it had a mean difference in FBS of 0.51, but, those with 36 months of follow-up had a mean difference in FBS of merely 0.07 (Fig. 3a). Similarly, this decline in mean FBS was noted for both genders, but not men nor women separately (Fig. 3b), for both adults and children but particularly children (Fig. 3c), and for those studies with a theoretical framework (Fig. 3d).

## Discussion

Based on our results, we found that studies testing for educational interventions against diabetes are few. Nevertheless, such interventions (number of participants = 16,106) can reduce diabetes incidence by 46.0%, along with causing favorable changes in the mean levels of FBS and A1c, BMI, and waist circumference. The interventions were effective for both genders, but men in particular, and the use of theory-based interventions. There was no effect of such interventions on both systolic and diastolic blood pressure, and the effect of interventions did not vary with the duration of follow-up. The most frequent theories used for interventions were social cognitive, t5 instructional design, health belief, and social marketing theories.

It was essential to conduct this study because diabetes has a huge affected and at-risk population. So, given the sheer size of the diabetes problem, primary prevention is the only way by which the public health burden from diabetes can be reasonably reduced or controlled. Moreover, many regions, such as ours, are showing a considerable rate of increase in diabetes than elsewhere. Thus, newer empirical evidence about the primary prevention of diabetes are needed that may help to restrain the growing burden of diabetes [2].

We targeted community-based educational interventions. It is now well-established that the health education of the public is a cardinal tool for both disease prevention and health promotion [7]. This is concordant with the findings of this study where diabetes incidence reduced by 46.0% overall. Health education works by bringing changes at both the cognitive, affective, and behavioral level [7] that may aid in disease prevention and control. The effect of educational interventions was more profound on FBS than A1c (Figs. 2 and 3). This lack of adequate reduction in A1c could be related to methodological aspects (e.g., inadequate power). For example, while the number of studies and sample size for FBS was 11 and 11,578, these were 6 and 6,416 in case of A1c (Supple file 2). Also, the smaller improvement in A1c could be related to the fact that the participants could be more acquainted with normal glucose level than A1c. The other factors associated with A1c may include possible genetic determination of A1c [5, 6] than FBS [40]. Also, A1c thresholds are not uniform worldwide, and the sensitivity of these thresholds differ in different racial groups [41, 42]. There are also different laboratory assay methods that may affect the measurement accuracy of A1c [43]. Moreover, A1c levels differ with iron-deficiency anemia [44], a factor with a varying frequency between populations. Accumulating evidence also suggest preventive impact of doing yoga in high-risk populations i.e. those with pre-diabetes and/or metabolic syndrome [28].

Besides FBS and A1c, the favorable impact of educational interventions was noted in waist circumference and BMI (Fig. 2), which means that the participants changed in central obesity [45]. This is a good outcome because waist circumference is a better predictor of the development of diabetes than BMI [46]. The maximum change was noted in a study that implemented educational intervention through community health workers (Fig. 2d). It is logical to expect a higher probability of success in educational interventions if necessary social support, communication, monitoring and feed back to patients are provided by the intervener(s) [47]. The identified educational interventions in this study have not indicated a significant effect on sBP and dBP probably because of the age range of participants and therefore, not considering them to be hypertensive or being at risk of high blood pressure in the conducted studies. Insignificant association of the implemented educational interventions and BP levels could perhaps be related to the applied methodology (e.g., undesirable statistical power) rather than an incongruence effect. However, others have shown that individuals may have an extremely poor attitude towards BP control, which needs to be scrutinized in separate independent interventions. For example, the prescribed therapeutic regimens are believed by some people to prohibit life’s pleasure and control personal liberty or freedom of choice [48, 49].

### Subgroup analyses

We performed various subgroup analyses with regards to the effect of interventions on FBS vis-à-vis the duration of follow-up, gender, age, and theoretical framework (Figs. 1, 2, 3). There was no association of the duration of follow-up with the change in FBS levels. These results may perhaps be related to the degree of attendance or intensity of intervention sessions. For instance, the studies with a longer follow-up showed a lower reduction in FBS, which could be related to pathophysiological progression toward diabetes; perhaps meaning that the suitability of educational interventions may vary with the pathophysiological progression towards the development of diabetes [30]. The longer programs have other impediments such as competing demands at home, not adequately motivated, no accompanying companion, and environmental factors such as bad weather. Others have also shown that the benefits of physical activity interventions decrease with longer follow-up [50].

We found that the use of theoretical framework-based interventions brought more favorable changes in FBS levels. These results are not different from what we and others [16] anticipate that theories may provide a better model-fit with diabetes prevention [16]. However, in contrast to the theory-based paradigm, others have been seemingly critical of the effectiveness of theory-based interventions [51]. But, that is because the data supporting theory-based paradigms are not adequate yet, and even their conclusions are limited by methodological and reporting issues [52]. We agree with the reasoning of the current lack of adequate data as only a few (*n* = 8, 40.0%) studies had a predefined theoretical framework for their intervention in our study. Studies without a theoretical framework had also shown their effectiveness in reducing diabetes risk parameters in our study. However, the possibility of better effectiveness with theory-based interventions remains open until adequate newer data becomes available.

We found that only 19 of 8181 studies had looked at health education as their intervention for the reduction of diabetes risk parameters. Even more unfortunate was the fact that the majority (*n* = 12, 63.1%) of these studies were conducted in merely three high-income countries. From our region, we found only one study conducted in our country, thereby leaving aside most of the other countries. Thus, there is no surprise that the goal of reducing the burden of diabetes remains lagged [53]. In terms of bibliometric efficiency, it is also noteworthy that only 19 studies were obtained from 8181 studies (0.2%), which shows a misfit rate of 99.7% (Fig. 1 and suppl file 1). So, from these results, one may surmise that databases are a cumbersome and imprecise source of literature, leading to unwarranted loss of precious time and labor. The associated reasons could perhaps be poor indexing since index terms and search keywords were carefully selected after a limited search of MEDLINE and EMBASE.

Although the effects were non-significant, FBS was found to be decreased more prominently for men than women (Fig. 3b). There can be numerous reasons for this difference. For example, the most prominent risk factor of diabetes is obesity, which is more common in women [10]. After that, there are diversities in biology, epigenetic mechanisms, culture, nutrition, lifestyle, environment, and socioeconomic status. Each of these may distinctively impact the differences in predisposition, development, and clinical presentation of diabetes between men and women. Furthermore, sex hormones have a great impact on energy metabolism, body composition, vascular function, and inflammatory responses, and women may have more unfavorable cardio-metabolic traits [11].

### Publication bias and heterogeneity

We found no indication of publication bias in our study. We detected heterogeneity, but that may only become evident after the data collection and analysis. For this reason, we used the random-effects analysis, which in part accounts for the heterogeneity between studies. However, being stricter with the inclusion criteria would have eliminated most of the studies, like those with a particular theoretical framework. Each research team prefers to use their ways to develop and report interventions [54].

### Limitations

Our work has some limitations. First, our study is post hoc, which means that we had to rely on the data that were available for us. Second, the number of studies was low; for instance, the number of published diabetes incidence studies was merely three, by the time of study search. This brings us to the necessary predicament that every health agency is interested in that the burden of diabetes reduces; yet, the effort is far from being the bare minimum for having the necessary fundamental epidemiological data on diabetes. Because of limits in available data, we could not compare the intended group analysis, such as between different theoretical frameworks. Third, we did not compare educational interventions with interventions for other kinds of diabetes. Fourth, heterogeneity was high, which is not unexpected. One way to reduce the heterogeneity between studies would be to use theoretical framework-based interventions that would then enhance the comparability between studies. Community-based educational interventions are often conducted under real-world conditions, so they are often with quasi-experimental designs and a high level of heterogeneity within samples [18]. That is why we included studies with both randomized control/ pseudo-randomized designs. Fifth, we included English language studies after 2000 till 2020 only, and our study did not evaluate feasibility outside the study settings. Lastly, few studies were of low quality, which may have affected some of our results.

### Conclusions and recommendations for research

Based on a comprehensive data collection of about 16,106 participants and reasonable analyses, we conclude that there is an acute paucity of reliable community-based educational interventions towards the prevention of diabetes from both high- and low-middle-income populations. Nevertheless, educational interventions may reduce diabetes incidence by 46.0%, particularly through effectuating a reduction in fasting blood glucose, body mass index, and waist circumference. The even more useful result is that diabetes risk parameters may reduce irrespective of the duration of follow-up, at as low as 6 months. Although both genders were found to experience a decline in diabetes risk parameters, men were most likely to benefit more than females. Such gender difference could be related to many possible reasons, including sex differences in diabetes or its risk factors across countries, diversity in epigenetic mechanisms, culture, lifestyle, sex hormones, body composition, vascular function, inflammatory responses, environment, and socioeconomic status between men and women. Finally, studies with theoretical framework-defined interventions were 58.0% more likely to effectuate favorable changes in diabetes risk parameters, particularly fasting blood glucose levels. Unfortunately, there is a lot left to discover and identify before we may eventually reach towards having efficacious and effective strategies for the prevention of diabetes. More research regarding sex-dimorphic pathophysiological mechanisms of T2DM and its complications could contribute to more personalized diabetes care in the future and would thus promote more awareness in terms of sex- and gender-specific risk factors.

## Supplementary Information

**Additional file 1.** Supplementary Data: file 1: The search strings for literature search.

**Additional file 2.** Supplementary Data: file 2: Characteristics of community-based educational interventions designed to prevent or delay Type 2 Diabetes.

## Data Availability

All data has been summarized and provided in the supplementary files.

## References

[1] Chisari G, Borzì AM, Chisari CG, C.E (2017). Amniotic membrane use in type 2 diabetes patients with chronic ulcers: microbiological evaluation and therapeutic benefits. Acta Med Mediterr.

[2] Shaw JE, Sicree RA, Zimmet PZ (2010). Global estimates of the prevalence of diabetes for 2010 and 2030. Diabetes Res Clin Pract.

[3] Esteghamati A (2017). Diabetes in Iran: prospective analysis from first nationwide diabetes report of National Program for Prevention and Control of Diabetes (NPPCD-2016). Sci Rep.

[4] Organization, W.H. Antimicrobial resistance: global report on surveillance: World Health Organization; 2014.

[5] Saaristo T (2010). Lifestyle intervention for prevention of type 2 diabetes in primary health care: one-year follow-up of the Finnish National Diabetes Prevention Program (FIN-D2D). Diabetes Care.

[6] Craig P (2013). Developing and evaluating complex interventions: the new Medical Research Council guidance.

[7] Chisanga C. Diabetes in young people in Africa: diabetes education, an important key element to managing diabetes in Zambia, in 13th European Diabetes and Endocrinology Congress, A. Diabetes Association of Zambia, Editor 2018. J Diabetes Metab Ireland. 2018. Available at: https://www.longdom.org/proceedings/diabetes-in-young-people-in-africa-diabetes-educationan-important-key-element-to-managing-diabetes-in-zambia-45567.html. Accessed 7 Mar 2021.

[8] Peyrot M, R.R (2011). Patient-reported outcomes in adults with type 2 diabetes using mealtime inhaled technosphere insulin and basal insulin versus premixed insulin. Diabetes Technol Ther.

[9] Baquedano IR, Martins TA, Zanetti ML, S.M. (2010). Self-care of patients with diabetes mellitus cared for at an emergency service in Mexico. Rev Lat Am Enfermagem.

[10] Rankin P (2012). Effectiveness of a volunteer-delivered lifestyle modification program for reducing cardiovascular disease risk factors. Am J Cardiol.

[11] Werfalli M, et al. Effectiveness of community-based peer-led diabetes self-management programmes (COMP-DSMP) for improving clinical outcomes and quality of life of adults with diabetes in primary care settings in low and middle-income countries (LMIC): a systematic review and meta-analysis. BMJ Open. 2015;5(7). 10.1136/bmjopen-2015-007635 PMC4513536 26179646

[12] Nissinen A, Berrios X, Puska P (2001). Community-based noncommunicable disease interventions: lessons from developed countries for developing ones. Bull World Health Organ.

[13] Shirinzadeh M (2019). The effect of community-based programs on diabetes prevention in low-and middle-income countries: a systematic review and meta-analysis. Glob Health.

[14] Renders CM (2001). Interventions to improve the management of diabetes in primary care, outpatient, and community settings. Diabetes Care.

[15] Weiss CH (1997). How can theory-based evaluation make greater headway?. Eval Rev.

[16] Michie S, Prestwich A (2010). Are interventions theory-based? Development of a theory coding scheme. Health Psychol.

[17] Karimy M, Zareban I, Taher M, Abedi A, A.M. (2016). Determinants of adherence to self-care behavior among women with type 2 diabetes: an explanation based on health belief model. Med J Islam Repub Iran.

[18] Galaviz KI (2018). Global diabetes prevention interventions: a systematic review and network meta-analysis of the real-world impact on incidence, weight, and glucose. Diabetes Care.

[19] Ali MK, Echouffo-Tcheugui JB, Williamson DF (2012). How effective were lifestyle interventions in real-world settings that were modeled on the Diabetes Prevention Program?. Health Aff.

[20] Mudaliar U (2016). Cardiometabolic risk factor changes observed in diabetes prevention programs in US settings: a systematic review and meta-analysis. PLoS Med.

[21] Balagopal P (2008). A community-based diabetes prevention and management education program in a rural village in India. Diabetes Care.

[22] Ibrahim N (2016). Effects of a community-based healthy lifestyle intervention program (co-HELP) among adults with prediabetes in a developing country: a quasi-experimental study. PLoS One.

[23] Rowan CP (2016). Community-based culturally preferred physical activity intervention targeting populations at high risk for type 2 diabetes: results and implications. Can J Diabetes.

[24] Penn L, Ryan V, White M (2013). Feasibility, acceptability and outcomes at a 12-month follow-up of a novel community-based intervention to prevent type 2 diabetes in adults at high risk: mixed methods pilot study. BMJ Open.

[25] Katula JA (2011). One-year results of a community-based translation of the Diabetes Prevention Program: Healthy-Living Partnerships to Prevent Diabetes (HELP PD) Project. Diabetes Care.

[26] Ackermann RT (2011). Long-term effects of a community-based lifestyle intervention to prevent type 2 diabetes: the DEPLOY extension pilot study. Chronic Illn.

[27] Ockene IS (2012). Outcomes of a Latino community-based intervention for the prevention of diabetes: the Lawrence Latino Diabetes Prevention Project. Am J Public Health.

[28] Daniel M (1999). Effectiveness of community-directed diabetes prevention and control in a rural Aboriginal population in British Columbia, Canada. Soc Sci Med.

[29] Raman A (2010). Insulin resistance is improved in overweight African American boys but not in girls following a one-year multidisciplinary community intervention program. J Pediatr Endocrinol Metab.

[30] Balagopal P (2012). A community-based participatory diabetes prevention and management intervention in rural India using community health workers. Diabetes Educ.

[31] Ramachandran A, Snehalatha C, Mary S, Mukesh B, Bhaskar AD, Vijay V (2006). The Indian Diabetes Prevention Programme shows that lifestyle modification and metformin prevent type 2 diabetes in Asian Indian subjects with impaired glucose tolerance (IDPP-1). Diabetologia.

[32] Harati H (2010). Reduction in incidence of type 2 diabetes by lifestyle intervention in a middle eastern community. Am J Prev Med.

[33] Group, D.P.P.R (2009). 10-year follow-up of diabetes incidence and weight loss in the Diabetes Prevention Program Outcomes Study. Lancet.

[34] Davies MJ (2016). A community based primary prevention programme for type 2 diabetes integrating identification and lifestyle intervention for prevention: the Let’s Prevent Diabetes cluster randomised controlled trial. Prev Med.

[35] Yin Z (2018). Cultural adaptation of an evidence-based lifestyle intervention for diabetes prevention in Chinese women at risk for diabetes: results of a randomized trial. Int Health.

[36] Pedley CF (2018). The 24-month metabolic benefits of the healthy living partnerships to prevent diabetes: a community-based translational study. Diabetes Metab Syndr Clin Res Rev.

[37] Sranacharoenpong K, Praditsorn P, Churak P (2018). Developing a diabetes prevention education program for community health care workers in Thailand: translation of the knowledge to at-risk people. J Public Health.

[38] Soltero EG (2019). ¡ Viva Maryvale!: a multilevel, multisector model to community-based diabetes prevention. Am J Prev Med.

[39] Soltero EG (2018). Effects of a community-based diabetes prevention program for Latino youth with obesity: a randomized controlled trial. Obesity.

[40] Yang Q (2010). Racial/ethnic differences in association of fasting glucose–associated genomic loci with fasting glucose, HOMA-B, and impaired fasting glucose in the US adult population. Diabetes Care.

[41] Aidenloo NS, et al. Optimal glycemic and hemoglobin a1c thresholds for diagnosing diabetes based on prevalence of retinopathy in an Iranian population. Iran Red Crescent Med J. 2016;18(8). 10.5812/ircmj.31254 PMC5065709 27781118

[42] Lim W, Ma S, Heng D, Tai ES, Khoo CM, Loh TP (2018). Screening for diabetes with HbA1c: test performance of HbA1c compared to fasting plasma glucose among Chinese, Malay and Indian community residents in Singapore. Sci Rep.

[43] Little RR, Roberts WL (2009). A review of variant hemoglobins interfering with hemoglobin A1c measurement.

[44] Coban E, Ozdogan M, Timuragaoglu A (2004). Effect of iron deficiency anemia on the levels of hemoglobin A1c in nondiabetic patients. Acta Haematol.

[45] Kamel E, McNeill G, Van Wijk M (2000). Change in intra-abdominal adipose tissue volume during weight loss in obese men and women: correlation between magnetic resonance imaging and anthropometric measurements. Int J Obes.

[46] Seo D-C, Choe S, Torabi MR (2017). Is waist circumference ≥ 102/88 cm better than body mass index ≥ 30 to predict hypertension and diabetes development regardless of gender, age group, and race/ethnicity? Meta-analysis. Prev Med.

[47] Khaylis A (2010). A review of efficacious technology-based weight-loss interventions: five key components. Telemed E Health.

[48] Nafradi L (2016). Intentional and unintentional medication non-adherence in hypertension: the role of health literacy, empowerment and medication beliefs. J Pub Health Res.

[49] Nations M (2011). Balking blood pressure “control” by older persons of Bambuí, Minas Gerais State, Brazil: an ethno-epidemiological inquiry. Cadernos Saúde Públ.

[50] Najafipour F, M.M, Yavari A, Nadrian H, Aliasgarzadeh A, Abbasi NM, Niafar M, Gharamaleki JH, Sadra V. Effect of regular exercise training on changes in HbA1c, BMI and VO2max among patients with type 2 diabetes mellitus: an 8-year trial. BMJ Open Diabetes Res Care. 2017;5(1). 10.1136/bmjdrc-2017-000414 PMC5687538 29177050

[51] Pals RA, Velasco ER, Grabowski D, S.T (2020). The role of theories in interventions targeting preteens with type 1 diabetes: a critical literature review. Child Care Health Dev.

[52] Dalgetty R, Dombrowski SU, M.C (2019). Examining the theory-effectiveness hypothesis: a systematic review of systematic reviews. Br J Health Psychol.

[53] Al-Maskari F, El-Sadig M, Nagelkerke N (2010). Assessment of the direct medical costs of diabetes mellitus and its complications in the United Arab Emirates. BMC Public Health.

[54] Schueller SM (2014). Purple: a modular system for developing and deploying behavioral intervention technologies. J Med Internet Res.

