# Supplementary material for: Community-based educational interventions for prevention of type II diabetes: a global systematic review and meta-analysis
Source: Syst Rev. 2021 Mar 20;10:81. doi: 10.1186/s13643-021-01619-3 (PMC7980624; doi:10.1186/s13643-021-01619-3)
Supplement: Supplementary file 1 — Additional file 1. Supplementary Data: file 1: The search strings for literature search. [file 13643_2021_1619_MOESM1_ESM.docx]

**Supplementary Data: file 1:** The search strings for literature search

| **Population** | **Intervention** | **Study design** | **Outcome** |
| --- | --- | --- | --- |
| “people at-risk for T2DM”  [Title/Abstract] | “community-based” [Title/Abstract] | “randomized controlled trial” [Title/Abstract] | “prevention” [Title/Abstract] |
| “ general populations”  [Title/Abstract] | “community based” [Title/Abstract] | “controlled clinical trial”[Title/Abstract] | “HbA1C level*”  [Title/Abstract] |
| “high-risk population*” [Title/Abstract] | education* AND intervention* [Title/Abstract] | “cross-over trial” [Title/Abstract] | “quality of life” [Title/Abstract] |
| “non-diabetic population*” [Title/Abstract] | education* AND program* [Title/Abstract] | “clinical trial” [Title/Abstract] | “health status” [Title/Abstract] |
| “pre-diabetic populations”  [Title/Abstract] | theory* AND education* AND intervention* [Title/Abstract] | “pre-experimental” [Title/Abstract] | “self-care behave*” [Title/Abstract] |
| “Diabetes Mellitus, Type 2” [Title/Abstract] | model* AND education* AND intervention* [Title/Abstract] | “experimental” [Title/Abstract] | “self care behave*” [Title/Abstract] |
| “diabetes type 2” [Title/Abstract] | lifestyle AND intervention* [Title/Abstract] | “quasi-experimental” [Title/Abstract] | “self-management behave*” [Title/Abstract] |
| “type 2 diabetes” [Title/Abstract] | lifestyle AND program* [Title/Abstract] | “randomized field trial” [Title/Abstract] | “self management behave*” [Title/Abstract] |
| “Diabetes Mellitus, Type 2” [Title/Abstract] | lifestyle AND education* [Title/Abstract] | “randomized clinical trial” [Title/Abstract] | “knowledge” [Title/Abstract] |
| “Diabetes Mellitus, Type 2” [Title/Abstract] | patient* AND education* [Title/Abstract] |  | “attitude” [Title/Abstract] |
| “high-risk individual*” [Title/Abstract] | program* AND evaluation* [Title/Abstract] |  | “self-efficacy”  [Title/Abstract] |
|  | education* approach [Title/Abstract] |  | “physical activity”  [Title/Abstract] |
|  | behavior* AND education [Title/Abstract] |  | “diet control”  [Title/Abstract] |
|  | behavior* AND approach [Title/Abstract] |  | “blood glucose”  [Title/Abstract] |
|  | “community intervention” [Title/Abstract] |  | “medical adherence”[Title/Abstract] |
|  | “community-based participatory research” [Title/Abstract] |  | “medication” [Title/Abstract] |
|  | “school-based” [Title/Abstract] |  | “weight control” [Title/Abstract] |
|  | “society-based” [Title/Abstract] |  | “program engagement”  [Title/Abstract] |

**Search string for PubMed**

(((((((((“clinical trial” [Title/Abstract]) OR “randomized” [Title/Abstract]) OR “cross over trial”[Title/Abstract]) OR “cross-over trial” [Title/Abstract]) OR “controlled clinical trial” [Title/Abstract]) OR “randomized controlled trial” [Title/Abstract] OR “experimental” [Title/Abstract] OR “pre-experimental” [Title/Abstract] OR “quazi-experimental” [Title/Abstract])) AND (((((((((((((((((prevention[Title/Abstract]) OR ((behavior change[Title/Abstract]) AND behavior change[Title/Abstract])) OR ""Patient Education as Topic""[Mesh]) OR ""Program Evaluation""[Mesh]) OR (((lifestyle program [Title/Abstract] OR lifestyle program[Other Term])) OR (life style program [Title/Abstract] OR life style program[Other Term]))) OR (((lifestyle intervention[Title/Abstract] OR lifestyle intervention [Other Term])) OR (life style intervention[Title/Abstract] OR life style intervention [Other Term]))) OR (((lifestyle education[Title/Abstract] OR lifestyle education[Other Term])) OR (life style education[Title/Abstract] OR life style education[Other Term]))) OR ((education* approach [Title/Abstract] OR education* approach[Other Term]))) OR ((education* intervention [Title/Abstract] OR education* intervention[Other Term]))) OR ((education* program [Title/Abstract] OR education* program[Other Term]))) OR (((behavior* education [Title/Abstract]) OR behavior* education[Other Term]))) OR (((behavior* Intervention [Title/Abstract]) OR behavior* Intervention[Other Term]))) OR ((behavior* program [Title/Abstract]) OR behavior* program[Other Term]))) AND (((""Diabetes Mellitus, Type 2"" [Mesh]) OR ((diabetes type 2[Title/Abstract]) OR diabetes type 2[Other Term])) OR ((type 2 diabetes [Title/Abstract]) OR type 2 diabetes[Other Term]))) AND ((((((((((community intervention [Title/Abstract]) OR community intervention[Other Term])) OR ""Community-Based Participatory Research""[Mesh]) OR ((Community-Based[Title/Abstract]) OR Community-Based [Other Term])) OR Community-Based[Text Word]) OR ((school based [Title/Abstract]) OR school Based[Other Term])) OR ((school-based[Title/Abstract]) OR school-based [Other Term])) OR ((((society based[Title/Abstract]) OR society based[Other Term]) OR social based [Title/Abstract]) OR social based[Other Term])) OR ((community[Title/Abstract]) OR community [Other Term])) [MeSH]))).

**Search string for ProQuest**

[(su(diabetes type 2) OR ab(diabetes type 2) OR ti(diabetes type 2)) AND ((ab(school based) OR ti(school based)) OR (ab(Community Based) OR ti(Community Based)) OR (ab(Community-Based) OR ti(Community-Based)) OR (ab(community) OR ti(community))) AND ((ab(Intervention) OR ti(Intervention)) OR (ab(behavior* Intervention) OR ti(behavior* Intervention)) OR (ab(education* program) OR ti(education* program)) OR (ab(education* intervention) OR ti(education* intervention)) OR (ab(education* approach) OR ti(education* approach)) OR (ab(lifestyle education) OR ti(lifestyle education)) OR (ab(lifestyle intervention) OR ti(lifestyle intervention)) OR (ab(lifestyle program) OR ti(lifestyle program)) OR (ab(Program Evaluation) OR ti(Program Evaluation)) OR (ab(behavior change) OR ti(behavior change)) OR OR (su(prevention) OR ab(prevention) OR ti(prevention))) AND pd(20000101-20191231)](https://search.proquest.com/myresearch/savedsearches.checkdbssearchlink:rerunsearch/1368380/SavedSearches?t:ac=SavedSearches).

**Search string for CINAHL**

(AB (“general population” OR (“high-risk individuals” OR “at-risk people” AB “pre-diabetic population” AND (AB “community-based” OR AB “community based” AND (AB education* AND intervention* OR AB education* AND program* OR AB theory* AND education* AND intervention* OR AB model* AND education* AND intervention* AND (AB “randomized controlled trial” OR AB “controlled clinical trial” OR AB “cross-over trial” OR AB “cross over trial” OR AB “randomized” OR AB “clinical trial” OR AB “experimental” OR AB “pre-experimental” OR AB “quazi-experimental”).

**Search string for EMBASE**

1. (Diabetes Mellitus, Type 2 or diabetes type 2 or type 2 diabetes or high-risk individual* or high-risk population* or non-diabetic population* or pre-diabetic population or general population).ab.

2. ((community intervention and community-based and community-based participatory research and society-based and society based and school-based and education* and intervention* and program*) or (theory* and education* and intervention* or model* and education* and intervention*) or (lifestyle and education* and lifestyle and intervention* and lifestyle and program* and education* approach) .ab.

3. (randomized controlled trial or controlled clinical trial or cross over trial or randomized or clinical trial or experimental trial or pre-experimental trial or quazi-experimental trial).ab.

4. high-risk for T2DM /

5. controlled clinical trial/ or “randomized controlled trial (topic)”/

6. 1 or 4

7. 3 or 5

8. 2 and 6 and 7

**Search string for the Cochrane Library**

1. Diabetes Mellitus, Type 2 or diabetes type 2 or type 2 diabetes or high-risk individual* or high-risk population* or non-diabetic population* or pre-diabetic population or general population:ti,ab,kw (Word variations will be searched)

2. Community-based education or Community-based intervention or society-based education or society-based intervention or health education or health education program or patient education or teach or train or educat or educat intervention: ti,ab,kw (Word variations will be searched).

3. behavior education or behavior change or behavior change theory or behavior change model or lifestyle education or lifestyle intervention or behavior approach: ti,ab,kw (Word variations will be searched)

4. No. 2 OR no. 3

5. Prevention or diet control or quality of life or health status or medication adherence or self-care behavior or self care behavior or self-management behavior or self management behavior or physical activity or HbA1c level or blood glucose or program management or weight control or knowledge or attitude or self-efficacy: ti,ab,kw (Word variations will be searched)

6. No. 1 AND no. 2 AND no. 3 AND no. 5.
