# Supplementary material for: Community-based educational interventions for prevention of type II diabetes: a global systematic review and meta-analysis
Source: Syst Rev. 2021 Mar 20;10:81. doi: 10.1186/s13643-021-01619-3 (PMC7980624; doi:10.1186/s13643-021-01619-3)
Supplement: Supplementary file 2 — Additional file 2. Supplementary Data: file 2: Characteristics of community-based educational interventions designed to prevent or delay Type 2 Diabetes. [file 13643_2021_1619_MOESM2_ESM.docx]

**Supplementary Data: file 2:** Characteristics of community-based educational interventions designed to prevent or delay Type 2 Diabetes

| **Reference** | **Setting, Target group, Sample Size (Intervention/ control),Theoretical framework** | **Age** | **Study design, duration** | **Aim** | **Primary/ Secondary outcome(s)** | **Intervention type** | | | **Control group** | **Community Involvement and culture components** | **Prevalence, incidence of diabetes** | **Response rate, Attrition rate, Follow –up (months)** |
| --- | --- | --- | --- | --- | --- | --- | --- | --- | --- | --- | --- | --- |
|  |  |  |  |  |  | Who | How | Where |  |  |  |  |
| Balagopal et al.(2008) | Rural India,  Rural community,703 (118 youths aged 10-17 years and 585 adults), None | 35.8 mean | Pre-post,  October 2002 to April 2003 | To evaluate community-based  lifestyle educational intervention | Change in FBG/BMI, bold pressure, knowledge on foods | Local graduates | Culturally and linguistically appropriate health education messages | Community | No control | The involvement of the village leaders, peer educators, and residents in planning and implementation phases of the project and served to highlight resources and requirements | 4.3, 12.1% | 74%  17%  6 months |
| Norliza et al. (2016) | Malaysian Sub- Urban communities, Adults with pre-diabetes, 268 (Ampangan community :122,32.9% , Senawang community :146,46.3%), HBM | 52.7 | Non-randomized controlled design, January 2012 to June 2013 | To evaluate lifestyle education intervention within community (CO-HELP) | Change in FBG, HBA1C, HDL and 2-hour plasma glucose/ Cholesterol levels, waist circumference, total cholesterol, diastolic BP, weight lost triglyceride, diet, and health-related quality of life | Local leaders and community volunteers | Individual counselling sessions, change behaviour sessions at the weekend in the community hall or recreational park, telephone calls or home visits, training  workshops | Community | Usual care group | The program was developed based on the needs and cultural sensitivity of the community. The education materials were refined and pretested on the community volunteers prior to delivery. | N R./ Influence after 12 months: 3 new cases in intervention versus 14 new cases in control | N.R,  At 6 months follow up: 0.8%,  At 12 months follow up: 9.1%,  12 months |
| Rowan et al. (2016) | Toronto, High risk of diabetes, 718, None | N.R | Pre-post longitudinal design, N.R. | To evaluate Community based culturally preferred physical activity intervention | Change in HbA1C/  oral glucose  tolerance test (OGTT), fasting glucose, fasting insulin, high-density lipoprotein-cholesterol (HDL-C), low density lipoprotein-cholesterol (LDL-C) insulin resistant (RI) | Culturally matched fitness instructors | Educational classes, dance and aerobic programs, traditional PA regimens, walking programs | Community centres  or churches | No control | Educational process took place in community health centres, religious centres and shopping malls and relied upon partnerships with community organizations, and a recognizable trusted relationship with community members. | N.R | 66%, 44%, 6 months |
| Penn et al.  (2013) | Central Middlesbrough, UK, 218, Supported by social marketing campaigns | 53.6 | Pre-post/ march 2009 to November 2010 | To assess the feasibility, acceptability & outcomes of a behavioural intervention | Change in PA levels & variety/  Change in weight & waist measurements & FINDRISK variables | NLNY trainers (potential participants and experienced local fitness trainers) | Behavioural counselling & cookery sessions, newsletter, online education, mobile text message, reflective discussion on PA, nutrition, & weight management | Leisure & Community | No control | The intervention design, name, logo and project documents were developed after consultation with potential participants and local fitness trainers | N.R. | 85.1%, 14.9%,  6 and 12 months |
| Katula et al.  (2011) | North American local society, High risk of diabetes, 301 ( 151 intervention, 150 control), None | 57.9 | Randomized controlled design/N.R. | To assess the impacts of a community-based translation of the DPP lifestyle weight loss (LWL) intervention | FBG/ insulin resistance, change weight, and anthropometric indicators | Community health workers | Group-based CHW-led sessions, personalized dietician counselling, DVD serves, newsletter, presentation from local community experts (e.g., YMCA, local grocery stores, and athletic foot ware stores) | Community sites (e.g., parks & recreational centres) | Enhanced usual care | Conducting the LWL intervention in community-based sites via a local DEP and CHWs, CHWs were community members with type 2 diabetes, well-controlled HBA1c, and history of healthy eating and physical activity. | N.R. | 92%, 1.1%,  6 and 12 months |
| Ackermann et al. (2011) | Indiana polis, YMCA facilities ,  Risk diabetes,  92 ( 46 intervention and 46 control),  None | 57.3 | Cluster-randomized controlled trial/  2006-2009 | To evaluate long-term effects of a lower-cost group-based adoption of DPP lifestyle intervention | Weight loss/ blood pressure, total cholesterol | Qualified YMCA instructors | Maintenance intervention for nutrition & PA educational sessions followed by monthly visits | YMCA facilities | Life style intervention with weekly (5), monthly (8) visits/  Brief advice for lifestyle change | Not discussed | Not reported | 70%, 28%,  16-24 months and 25-32 months |
| Ockene, et al. (2012) | Lawrence, Massachusetts,  High risk diabetes,  312 (162 intervention and 150 control cases),  Social cognitive theory & patient-centred counselling | 52 | Randomized controlled block design/ 2004-2006 | To assess the effectiveness of a community-based literacy sensitive and culturally tailored lifestyle intervention | Weight loss, HbA1C/ fasting lipid, blood pressure, FBS, dietary & PA assessment, QOL & depression scores | A team of 3 Spanish-speaking community individuals with post high-school education | Individual sessions, group sessions & home visits, building skills for dietary and physical activity | Participants’ homes and community site (the Lawrence senior centres) | Literacy sensitive and culturally tailored lifestyle intervention/ Usual care | Dietary advice based on Latino foods; targeting cultural beliefs and attitudes toward diabetes prevention; delivery of intervention in Spanish by bicultural and bilingual individuals from the community | N.R. | 94%, 6.8%,  1 year |
| Daniel et al.  (1999) | British Columbia rural Okanogan region,  Persons with or at familial risk for non-insulin-dependent  diabetes mellitus NIDDM,  (207),  Social learning theory | 46.5 | Quasi experimental non-equivalent control group design/  1994-1996 | To assess the effectiveness of a community-directed initiative in achieving & risk reduction or improved control amongst “high-risk” individuals with or at familial risk for diabetes | HbA1C/ BMI, Systolic blood pressure, Sweat-producing PA, Knowledge on diabetes | Indigenous health workers | aerobics and gentle exercise classes, walking group, health events, cooking demonstrations, smoking cessation group, supermarket and restaurant tours, development of skills for healthful living local newspaper, newsletters, media | Community | Behavioural change intervention environmental support for behavioural intervention/ two comparison communities | Pre intervention diagnostic efforts within community (community survey of systems community needs assessments & using media to empower community action | 16.1% | N.R, N.R  8, 16 and 24 months |
| Raman et al.  (2010) | Inner-city YMCA sites in Oakland,  Over weight African American children,  165 (87 intervention and 79 control cases),  Social cognitive theory | 9-11 years | Non randomized community intervention pilot study, May 2005 to May 2006 | To  assess effectiveness of community based lifestyle intervention program to reduce the risk for T2DM | Change in insulin resistance (HOMA- IR)/ change in weight, BMI, fasting glucose, fasting insulin | YMCA staff & instructors | Physical activity program (swimming, floor hockey), nutrition education (choosing healthy snacks, using low fat meat, using low-fat dairy) and dissection sessions, individual sessions online, advocating healthy eating, promoting healthy eating behaviours | Community | Lifestyle education (PA, nutrition & self-esteem promotion) / routine program with mailed information lifestyle modification | Consideration on cultural limitation & preferences of participant while designing & implementing the program | N.R | 66%, 38%,  One year |
| Balagopal et al. (2012) | Rural India,  Population, 1681,  None | 41.9 years mean | Pre-post,  December 2007 to May 2008 | To assess the effectiveness of a CBPR approach to diabetes prevention and management program | Change in FBG/ Ssystolic and diastolic blood pressure, Knowledge of diabetes and cardiovascular disease; general and abdominal obesity | Community health workers | Culturally & Linguistically appropriate health education messages | Community | Mixed, No control | Including the entire rural community (village elder), Pre planning community meetings before launching the program to build trust, confidence, and rapport with the stakeholders and academic partners. Black spokespersons were identified to work with the team. | Baseline prevalence 7.1% | 81.9, N.R,  6 months |
| Ramachanran et al. (2006) | Urban and rural India,  Risk group, 531 (group1control n=136, group2 LSM n=133, group3 MET n=133, group4 LSM+MET n=129), None | 35-55 years | Randomize controlled trial, N.R | To evaluate the impact of lifestyle modification on prevention of type 2 diabetes | Development of diabetes, indicated by either a FBG and /or 2-h oral glucose tolerance test | Physician, laboratory technician, social worker and helper | Lifestyle modification counselling through monthly telephone contact | Selected occupational setting | G1: Lifestyle modificati,G2: Metformin, G3: Lifestyle modification and Metformin; G4: standard care | N.R | The 3-years cumulative incidences of diabetes in lifestyle intervention group: 39.3% | 95.10%, NR,  36 months |
| Harati et al. (2010) | Urban Iran,  Population,  10368 (3931 intervention and 6437 control cases), None | 43 years mean | Pre-post design with control group,  1991-2005 | To assess the effect of school-based life style intervention | Change in FBG and 2-h oral glucose tolerance test | Community health volunteers | Nutrition education classes, PA & smoking cessation education, interventions & publications | Medical health centres | Nutritional education classes / patient without education | Participation in religious ceremonies; sessions in mosques particularly in the holy month of Ramadan; public conferences on occasions such as the world diabetes day or world no-Tabaco day contained health messages in lay language | Incidence rate: 12.2/1000 person-years and 8.2/1000 person-years, in the control and intervention groups, respectively | NA,  Intervention (43%) and control (41%),  3.6 years |
| Diabetes Prevention Program coordinating Centre  (2009) | Ethnic and racial minority group communities,  High- risk adults,  3819 (1079 intervention, 1073 metformin, and 1082 placebo cases), None | N.R | Randomized controlled trial,  September 2002 to August 2008 | To assess long-term effects of DPP on diabetes incidence | Development to diabetes, Change in FBG and 2-h oral glucose tolerance test/ weight loss, Blood pressure, plasma lipids, | N.R | Group-implemented lifestyle-intervention | Community | DPP, G1: lifestyle education classes /  G2: Metformin, G3: placebo | N.R | In lifestyle group diabetes incidence reduced by 34% (24–42) in the lifestyle group and 18% (7–28) in the metformin group compared with placebo | 88%, 18%,  5.7 years |
| Davies et al.  (2016) | Leicestershire, UK. ,  Prediabetes,  880  (443 intervention 447 control ) ,  Patient empowerment | 64, mean | Randomized controlled trial/ May 2009-july 2014 | To assess effectiveness of a structure education program targeting lifestyle and behaviour change | Development to diabetes/ HBA1c, LDL cholesterol, BMI, waist circumference, systolic blood pressure, knowledge, healthy behaviour, quality of life, and anxiety, | Trained educators | Let’s prevent program, which tailors the DESMOD structured education programme into a prevention context | Community | Control group / standard care group | The content and educational resources used within the programme were further tailored to the need of local south Asian population | N.R | 76%  /  24%  /  3years |
| Yin et al.  (2018 ) | Non-metropolitan communities china ,  Women at risk diabetes,  184(intervention group n=109, comparison group n= 75),  None | 51.96y(sd7.22) | Randomized controlled trial / may 2013 to November 2014 | To assess the feasibility & effectiveness of on evidence –based diabetes prevention program | HbA1c, weight, BMI, waist circumference, fasting glucose, heart rate /PA dietary quality score | Community health educators | Educational sessions nutrition, PA, behavioural monitoring, small-group session for goal setting and evaluation, counselling and social support | CHCs and health screening centre | Group-based lifestyle intervention built on the DPP group lifestyle balance program / General healthy lifestyle education without specific counselling or reinforcement | Intervention activities were adopted to address difference in lifestyle and cultural traditional values associated with food, cooking practices, resources available and healthcare practices in china. Modify the nutritional messages and recommendations based on the chines food guide pagoda | N.R | 95% at 6 months & 86% at 12 month  /  5%  /  6and 12 months |
| Pedley et al. (2017) | Midsized city in north Carolina USA,  Overweight & obese individuals ,  301)enhance usual care n= 150, group based lifestyle intervention n=151),  None | 58years mean | Randomized control trial / 2007-2011 | To compare the 12 and 24 month prevalence of metabolic syndrome in the two treatment arms of HELP PD. | Waist circumference male ≥102 cm (40in) female ≥88cm(35in);fasting triglycerides ; HDL cholesterol male (<40mg/dl) and female (<50mg/dl) or drug treatment for low HDL cholesterol ; blood pressure ≥130systolic or ≥85 diastolic or drug treatment for elevated blood pressure and fasting plasma glucose | Community health workers , nutritionist, | Lifestyle weight loss (LWL) ssessions with nutritionists, group sessions weekly, phone, monthly newsletter, | Single care centre | Group-based lifestyle intervention / enhanced care group | N.R | N.R | 92%  /  18%  /  12and 24mounth |
| Sranacharoenpong et al. (2018) | Semi-urban or rural districts, Thailand,  People at risk for diabetes, 987 (511 intervention, 476 control),  T5 instructional design model | Older than 50 | Randomized controlled design /N.R | To evaluate the effects of an education program for community health workers (CHCWs) on the knowledge, awareness and understanding of key message to healthy eating among community members at risk for diabetes | CHCWs Knowledge on diabetes education prevention/ body weight, body mass index, waist circumference and systolic blood pressure, diabetes risk score (DRS) | Community Health Care Workers (CHCWs) | E-learning sessions, problem-based learning, community-based application assignments, self-evaluations and on-line support | Health care centre | No training/ support | Implantation of the learning benefited from CHCWs' links to their specific communities and culture, and drew on their experience in other aspects of community health promotion | N.R | 80.5%, ~ 20%, 8 months |
| Soltero et al. (2019) | Latino community in phoenix, Arizona,  High risk Latino families with an obese child ,  58 families (prevention= 59 ,children=68 ),  Social cognitive theory | Adults 37.3 , children 9.8 | Pre-post design /2015-2018 | To Test the preliminary efficacy of a culturally grounded, diabetes prevention program for high-risk Latino families delivered through an IRPP | Weight, BMI, waist circumference, body fat, HBA1c & weight- specific QOL/ fat intake, fruit/ vegetable intake & PA | Community health workers, dietitians and health workers, | Orientation at the YMCA, enhancing self- efficacy, teaching goal setting, facilitating social support, emotional by addressing topic, such as self- esteem, family communication, roles, and responsibilities, nutrition session, exercise sessions | Academic research centre, park health centre, federally qualified health centres | Bilingual/bicultural registered dietitians and community health educators delivered nutrition education sessions to group families | Nutrition with educated and behavioural skills training (60 minutes, once/week ) and physical activity classes (60minut,three time/week) delivered at a YMCA  /  No control | N.R | 83%  /  N.R  /  2years |
| Soltero et al. (2018) | Latino –serving local YMCA, phoenix, Arizona, Obese youth  , 987 (511 intervention, 476 control), T5 instructional design model | 15.3 | Randomized control trial/ October 2012-august 2016 | To assess Short-and long-term effects of a community based lifestyle intervention | Insulin sensitivity, QOL, / total body fat & waist circumference | Community stakeholders, YMCA fitness instructors | Nutrition and health education, exercise, behaviour change strategies, enhancing self-efficacy for healthy lifestyle behaviours through goal-setting, vicarious experience, role modelling, and verbal encouragement , psychosocial consequence associated with Pedi metric obesity, | Latino-serving health clinical , local YMCA | Supporting through an academic-community collaboration that engaged an accredited diabetes education program from Latino-serving health clinic and local YMCA. Community stakeholder within the partnership have worked collaborating integrated whit Latino cultural values such as feminism | general information on healthy lifestyle behaviour | N.R | 82.5%  /  N.R  /  3,6, and 12 months |
